# The genome sequence of purple glasswort, *Salicornia ramosissima* Woods (Amaranthaceae)

**DOI:** 10.12688/wellcomeopenres.21552.1

**Published:** 2024-05-15

**Authors:** Sahr Mian, Maarten J. M. Christenhusz, Ilia J. Leitch, Andrew R. Leitch, Royal Botanic Gardens Kew Genome Acquisition Lab, Plant Genome Sizing collective, Darwin Tree of Life Barcoding collective, Wellcome Sanger Institute Tree of Life Management, Samples and Laboratory team, Wellcome Sanger Institute Scientific Operations: Sequencing Operations, Wellcome Sanger Institute Tree of Life Core Informatics team, Tree of Life Core Informatics collective, Darwin Tree of Life Consortium

**Affiliations:** 1 Royal Botanic Gardens Kew, Richmond, England, UK; 2 Curtin University, Perth, Western Australia, Australia; 3 Queen Mary University of London, London, England, UK

**Keywords:** Salicornia ramosissima, purple glasswort, genome sequence, chromosomal, Caryophyllales

## Abstract

We present a genome assembly from an individual *Salicornia ramosissima* (purple glasswort; Tracheophyta; Magnoliopsida; Caryophyllales; Chenopodiaceae). The genome sequence is 529.1 megabases in span. Most of the assembly is scaffolded into 9 chromosomal pseudomolecules. The mitochondrial and plastid genome assemblies have lengths of 328.55 kilobases and 153.3 kilobases in length, respectively.

## Species taxonomy

Eukaryota; Viridiplantae; Streptophyta; Streptophytina; Embryophyta; Tracheophyta; Euphyllophyta; Spermatophyta; Magnoliopsida; Mesangiospermae; eudicotyledons; Gunneridae; Pentapetalae; Caryophyllales; Chenopodiaceae; Salicornioideae; Salicornia; Salicornia subgen. *Salicornia*; *Salicornia ramosissima* Woods (NCBI:txid267548).

## Background

Glasswort or marsh samphire of the genus *Salicornia* L. is a salt-loving (halophytic) annual succulent, found along the coasts of most of the world, as well as inland in salt-flats and along salt lakes. In Britain and Ireland, it is typically found in salt marshes and along salty creeks and coastal mudflats. The genus is widely polymorphic, making the species difficult to distinguish ( Valdés & Castroviejo, 1990). Three main groups or aggregates can be distinguished in the UK, characterised by the numbers and sizes of flowers per group, and the shape and size of fertile segments and seeds ( Lopes *et al.*, 2023; Stace *et al.*, 2019).

**Figure 1.**
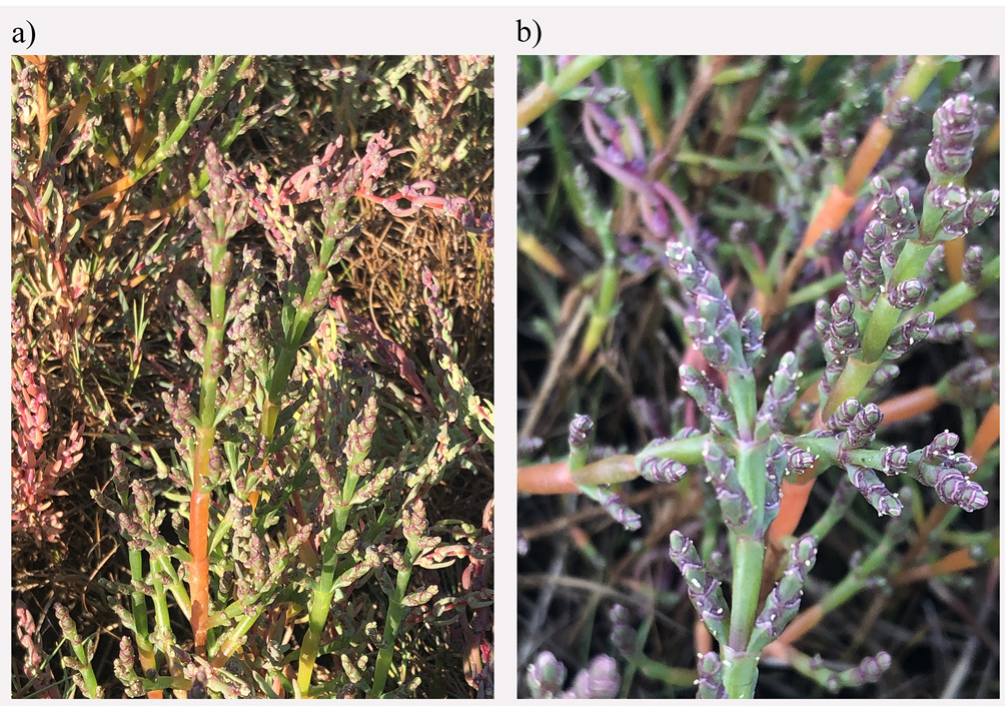
Photograph of the *Salicornia ramosissima* (dcSalRamo1) specimen used for genome sequencing.

*Salicornia ramosissima* belongs to the *S. europaea* L. aggregate, which may be recognised by groups of three flowers, short anthers (≤ 0.5 mm) on a single stamen, the central flowers much larger than the two laterals and fertile segments convex with a scarious border. It is called purple glasswort, because the plants appear tinged with reddish-purple in the sun when the season progresses ( Figure 1). It is restricted to Atlantic and North Sea coasts of Europe.

Like other halophytes, *Salicornia ramosissima* has high rates of mineral retention from the environment, and is rich in sodium and potassium ( Correia *et al.*, 2022). This probably contributed to the use of the species in glass and soap making during the 16 ^th^ century and gave rise to the origin of one of its common names - glasswort. For example, burning the plant tissue converted the sodium into soda ash (i.e., sodium carbonate), which was a key ingredient for glassmaking as it reduced the furnace temperature required to melt silica.

The origin of its other common name ‘samphire’ probably derives from St Peter, patron saint of fishermen ( Onions, 1966). However, it is noted that this common name is broadly used as a term for marine plants with succulent, edible juicy stems and leaves. Thus, apart from *Salicornia* and the related *Sarcocornia* A. J. Scott, ‘samphire’ has also been used to refer to the unrelated *Crithmum maritimum* L. (Apiaceae) commonly known as rock samphire and *Limbarda crithmoides* (L.) Dumort. (Asteraceae) which is often referred to as the golden samphire.

*Salicornia ramosissima* has been widely used for food, either fresh or pickled, often accompanying fish dishes. In recent years it has become popular in gourmet cuisine. Given its preference for salty, nutrient-rich environments, the nutritional value of the plant has been researched, and it is being investigated as a potential food source in famine situations ( Lopes *et al.*, 2023). Success with marsh samphire cultivation could add a new dimension to agriculture, helping to meet the needs of people and livestock in some of the driest, saltiest regions of the globe. The plant can be used as animal fodder and seeds can be harvested for high quality vegetable oil ( Clark, 1994). As the plant may be irrigated with seawater, the cultivation of *Salicornia* is especially attractive in areas where freshwater availability is limited.

There has been some debate over the chromosome number for this species as both diploids with 2n = 18 and tetraploids with 2n = 36 have been reported from British-collected material (e.g. Dalby, 1962; Hambler, 1954; Henniges *et al.*, 2022; Maude, 1939). While it has been suggested that this may be due to the challenges of distinguishing *S. ramoisissima* from the broader *S. europaea* aggregate, Dalby (1962) confirmed the presence of that both diploid and tetraploid cytotypes of *S. ramoisissima* in material collected from Britain.

The chromosome-level genome sequence presented here is the first for any species in the genus, but as additional species are sequenced, this will help in understanding the taxonomic diversity of the genus and help tease apart the species in the different species aggregates. In addition, the genome joins whole genome assemblies already publicly available for other halophytes in the related genus *Suaeda* (e.g. *S. aralocaspica*; Wang *et al*., 2019) and *S. glauca*; Cheng *et al.*, 2023). As temperatures and sea levels continue to rise, the need for crops that can grow where little else will and can withstand drought and an increasingly salinised and degraded environment will undoubtedly increase ( Lopes *et al.*, 2023). This genome will also help us to understand salt tolerance in *S. ramosissima* and related species.

## Genome sequence report

The genome was sequenced from a specimen of *Salicornia ramosissima* ( Figure 1) collected from Widewater Lagoon, Shoreham, West Sussex, UK (50.82, –0.30). Using flow cytometry, the genome size (1C-value) was estimated to be 0.65 pg, equivalent to 630.8 Mb/1C. A total of 77-fold coverage in Pacific Biosciences single-molecule HiFi long reads was generated. Primary assembly contigs were scaffolded with chromosome conformation Hi-C data. Manual assembly curation corrected 10 missing joins or mis-joins, reducing the scaffold number by 9.09%, and increasing the scaffold N50 by 3.18%.

**Figure 2.**
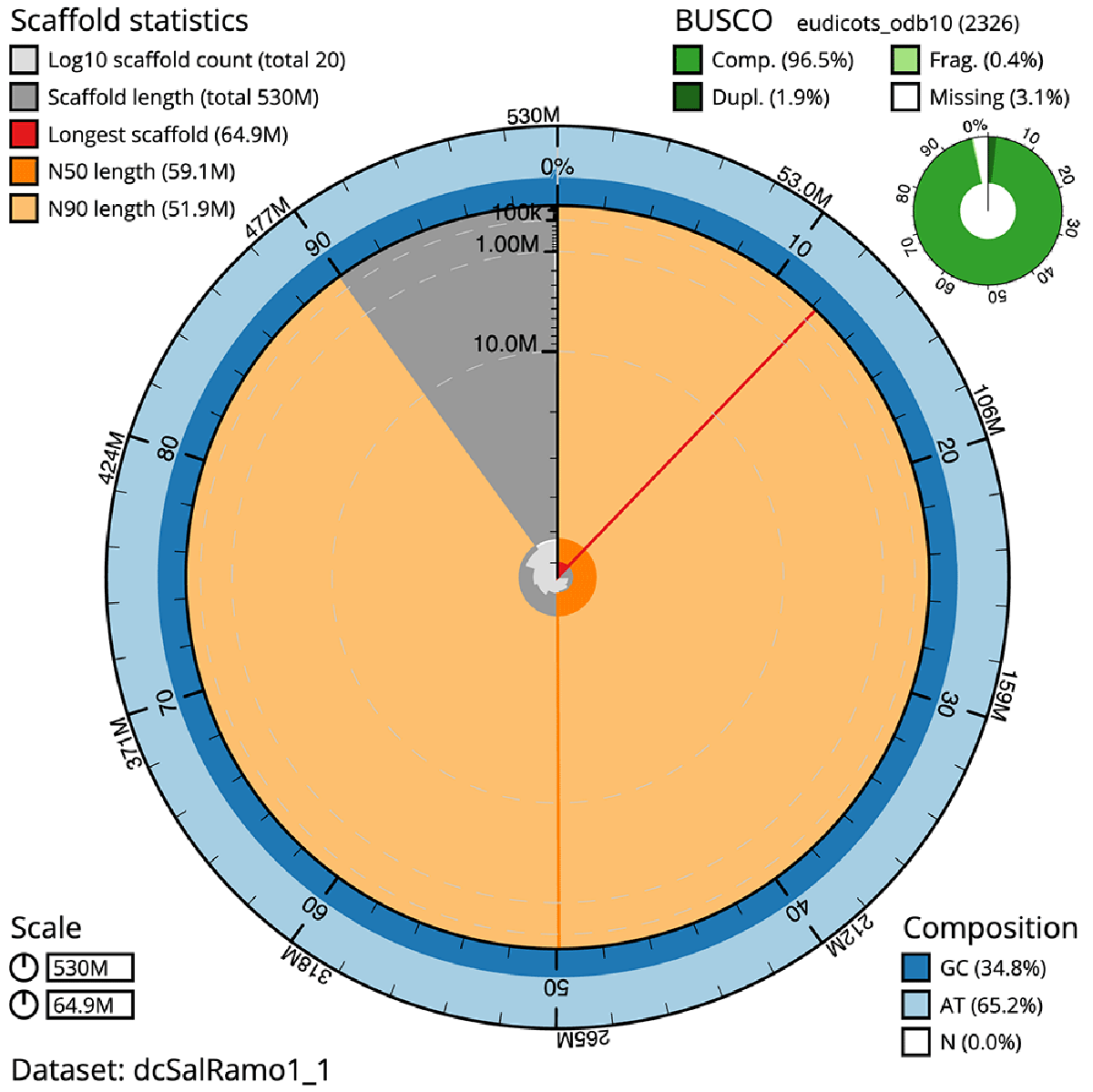
Genome assembly of *Salicornia ramosissima*, dcSalRamo1.1: metrics. The BlobToolKit Snailplot shows N50 metrics and BUSCO gene completeness. The main plot is divided into 1,000 size-ordered bins around the circumference with each bin representing 0.1% of the 529,593,104 bp assembly. The distribution of scaffold lengths is shown in dark grey with the plot radius scaled to the longest scaffold present in the assembly (64,877,190 bp, shown in red). Orange and pale-orange arcs show the N50 and N90 scaffold lengths (59,065,583 and 51,935,755 bp), respectively. The pale grey spiral shows the cumulative scaffold count on a log scale with white scale lines showing successive orders of magnitude. The blue and pale-blue area around the outside of the plot shows the distribution of GC, AT and N percentages in the same bins as the inner plot. A summary of complete, fragmented, duplicated and missing BUSCO genes in the eudicots_odb10 set is shown in the top right. An interactive version of this figure is available at https://blobtoolkit.genomehubs.org/view/dcSalRamo1_1/dataset/dcSalRamo1_1/snail.

**Figure 3.**
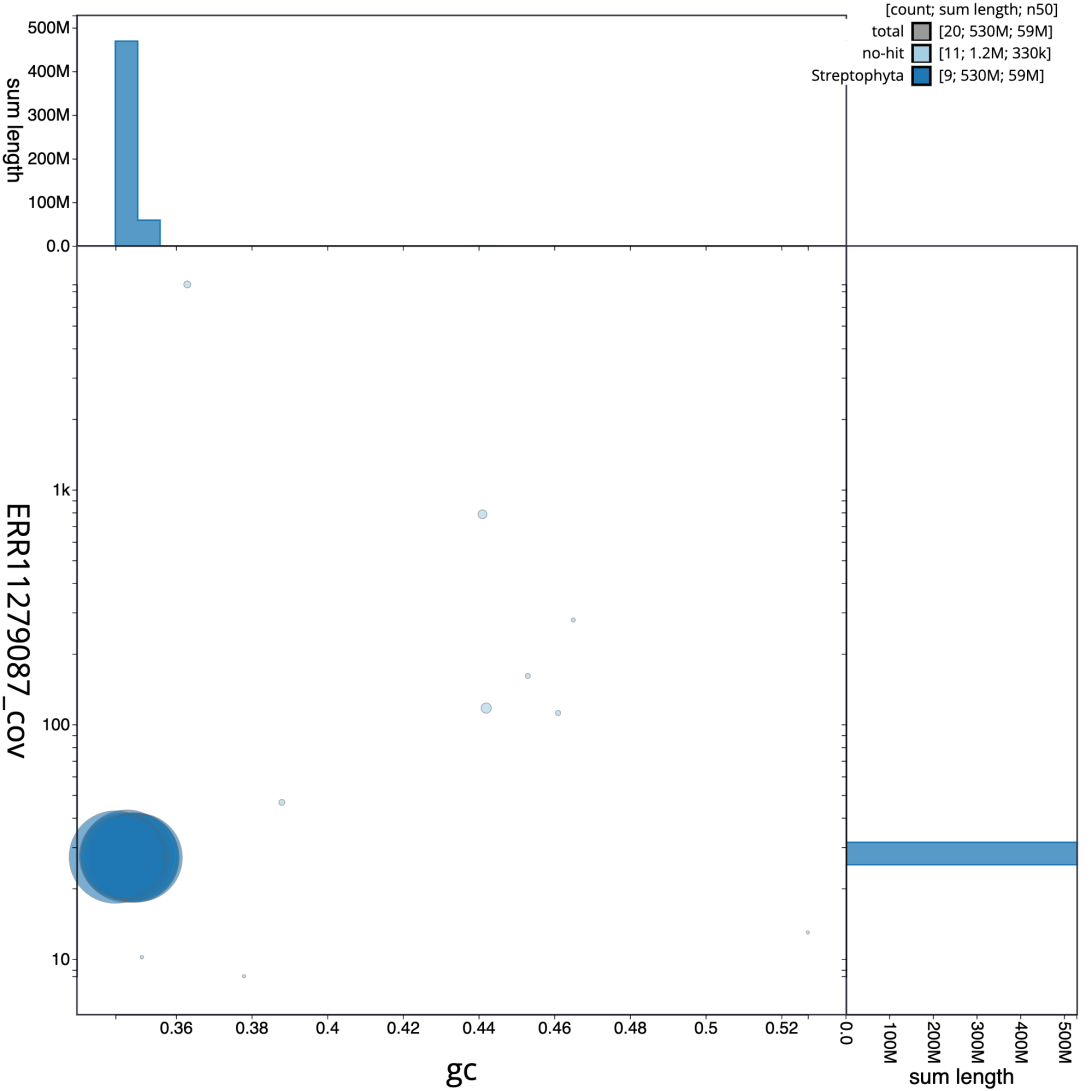
Genome assembly of *Salicornia ramosissima*, dcSalRamo1.1: BlobToolKit GC-coverage plot. Scaffolds are coloured by phylum. Circles are sized in proportion to scaffold length. Histograms show the distribution of scaffold length sum along each axis. An interactive version of this figure is available at https://blobtoolkit.genomehubs.org/view/dcSalRamo1_1/dataset/dcSalRamo1_1/blob.

**Figure 4.**
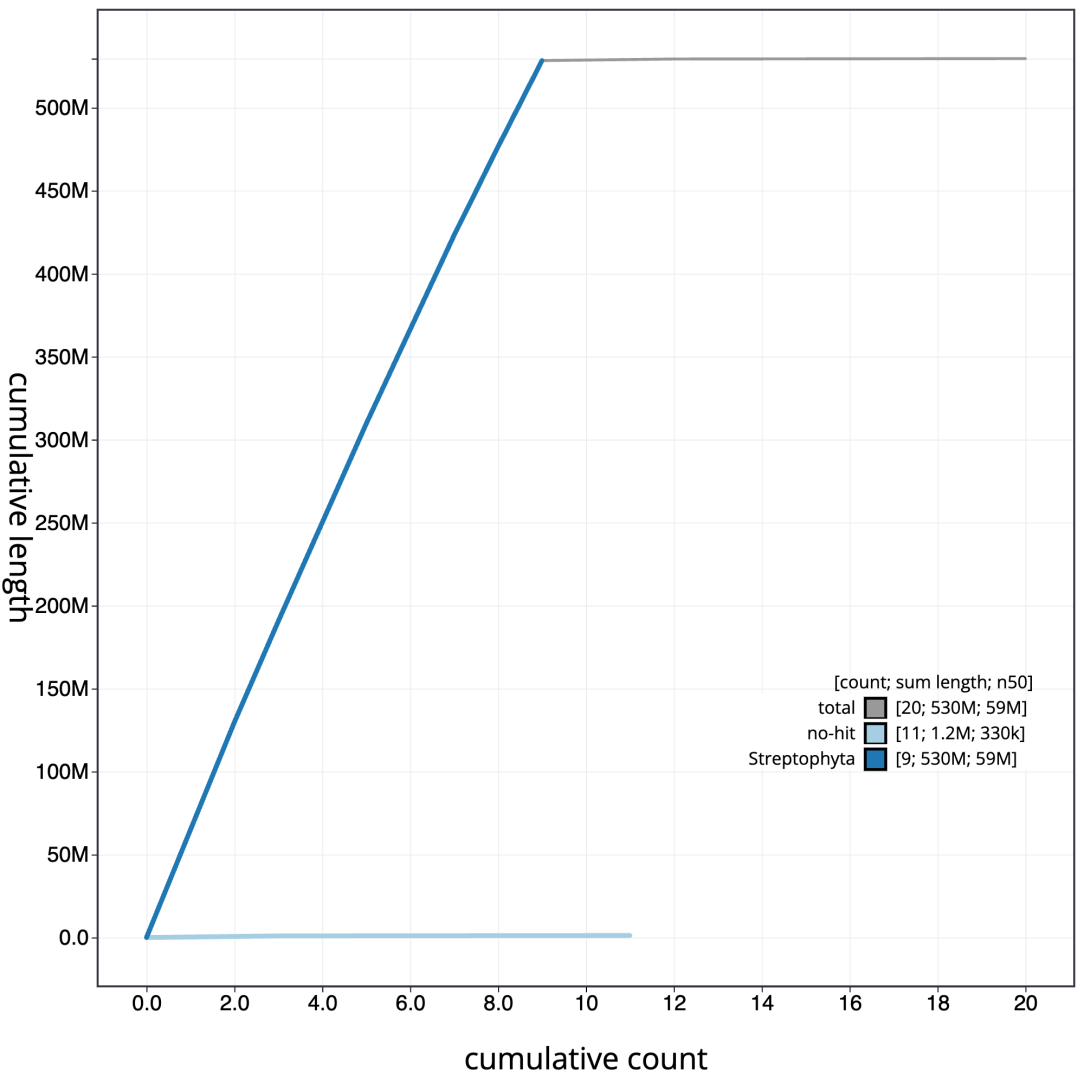
Genome assembly of *Salicornia ramosissima*, dcSalRamo1.1: BlobToolKit cumulative sequence plot. The grey line shows cumulative length for all scaffolds. Coloured lines show cumulative lengths of scaffolds assigned to each phylum using the buscogenes taxrule. An interactive version of this figure is available at https://blobtoolkit.genomehubs.org/view/dcSalRamo1_1/dataset/dcSalRamo1_1/cumulative.

**Figure 5.**
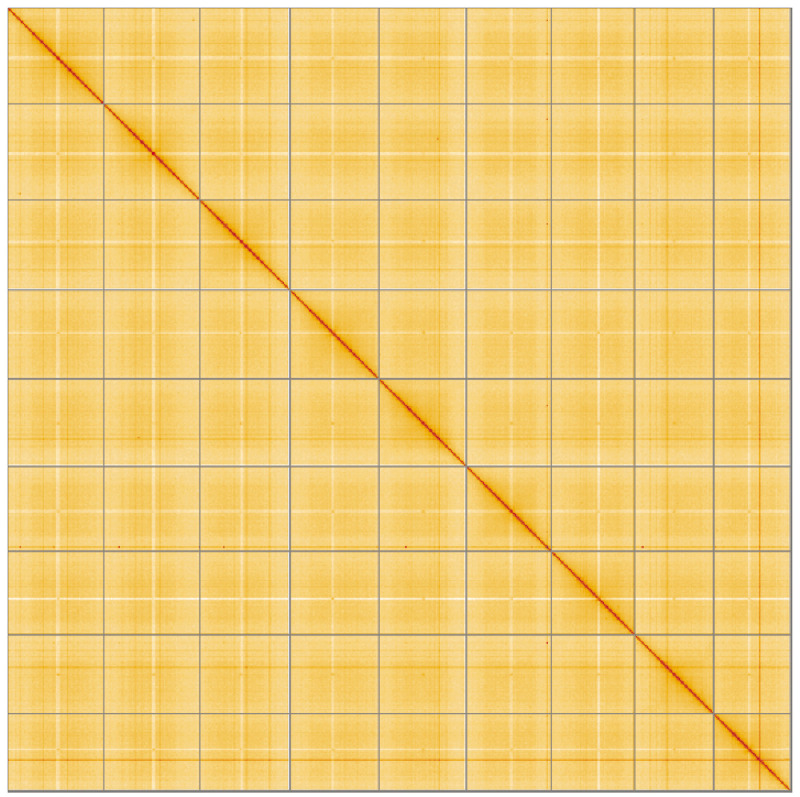
Genome assembly of *Salicornia ramosissima*, dcSalRamo1.1: Hi-C contact map of the dcSalRamo1.1 assembly, visualised using HiGlass. Chromosomes are shown in order of size from left to right and top to bottom. An interactive version of this figure may be viewed at https://genome-note-higlass.tol.sanger.ac.uk/l/?d=ah548Z1nSyeXnOhbeNhmMw.

The final assembly has a total length of 529.1 Mb in 18 sequence scaffolds with a scaffold N50 of 59.1 Mb ( Table 1). The snail plot in Figure 2 provides a summary of the assembly statistics, while the distribution of assembly scaffolds on GC proportion and coverage is shown in Figure 3. The cumulative assembly plot in Figure 4 shows curves for subsets of scaffolds assigned to different phyla. Most (99.77%) of the assembly sequence was assigned to 9 chromosomal-level scaffolds. Chromosome-scale scaffolds confirmed by the Hi-C data are named in order of size ( Figure 5; Table 2). While not fully phased, the assembly deposited is of one haplotype. Contigs corresponding to the second haplotype have also been deposited. The mitochondrial and plastid genomes were also assembled and can be found as contigs within the multifasta file of the genome submission.

**Table 1.** Genome data for *Salicornia ramosissima*, dcSalRamo1.1.

Project accession data		
Assembly identifier	dcSalRamo1.1	
Species	*Salicornia ramosissima*	
Specimen	dcSalRamo1	
NCBI taxonomy ID	267548	
BioProject	PRJEB61611	
BioSample ID	SAMEA10369832	
Isolate information	dcSalRamo1: leaf (DNA and Hi-C sequencing)	
Assembly metrics *		*Benchmark*
Consensus quality (QV)	71.4	*≥ 50*
*k*-mer completeness	100.0%	*≥ 95%*
BUSCO **	C:96.5%[S:94.6%,D:1.9%], F:0.4%,M:3.1%,n:2,326	*C ≥ 95%*
Percentage of assembly mapped to chromosomes	99.77%	*≥ 95%*
Sex chromosomes	None	*localised homologous pairs*
Organelles	Mitochondrial genome: 328.55 kb Plastid genome: 153.3 kb	*complete single alleles*
Raw data accessions		
PacificBiosciences SEQUEL II	ERR11279088, ERR11279087	
Hi-C Illumina	ERR11271531	
Genome assembly		
Assembly accession	GCA_951394345.1	
*Accession of alternate haplotype*	GCA_951394335.1	
Span (Mb)	529.1	
Number of contigs	143	
Contig N50 length (Mb)	6.6	
Number of scaffolds	18	
Scaffold N50 length (Mb)	59.1	
Longest scaffold (Mb)	64.88	

* Assembly metric benchmarks are adapted from column VGP-2020 of “Table 1: Proposed standards and metrics for defining genome assembly quality” from ( Rhie *et al.*, 2021). ** BUSCO scores based on the eudicots_odb10 BUSCO set using version 5.3.2. C = complete [S = single copy, D = duplicated], F = fragmented, M = missing, n = number of orthologues in comparison. A full set of BUSCO scores is available at https://blobtoolkit.genomehubs.org/view/dcSalRamo1_1/dataset/dcSalRamo1_1/busco.

**Table 2.** Chromosomal pseudomolecules in the genome assembly of *Salicornia ramosissima*, dcSalRamo1.

INSDC accession	Chromosome	Length (Mb)	GC%
OX596232.1	1	64.88	34.5
OX596233.1	2	64.87	34.5
OX596234.1	3	60.85	35.0
OX596235.1	4	59.9	35.0
OX596236.1	5	59.07	35.0
OX596237.1	6	57.34	34.5
OX596238.1	7	56.26	35.0
OX596239.1	8	53.29	34.5
OX596240.1	9	51.94	34.5
OX596241.1	MT	0.33	44.0
OX596242.1	Pltd	0.15	36.0

The estimated Quality Value (QV) of the final assembly is 71.4 with *k*-mer completeness of 100.0%, and the assembly has a BUSCO v5.3.2 completeness of 96.5% (single = 94.6%, duplicated = 1.9%), using the eudicots_odb10 reference set ( *n* = 2,326).

Metadata for specimens, barcode results, spectra estimates, sequencing runs, contaminants and pre-curation assembly statistics are given at https://links.tol.sanger.ac.uk/species/267548.

## Methods

### Sample acquisition, genome size estimation and nucleic acid extraction

A specimen of *Salicornia ramosissima* (specimen ID KDTOL10391, ToLID dcSalRamo1) was collected from Widewater Lagoon, Shoreham, West Sussex, UK (latitude 50.82, longitude –0.30) on 2021-09-07. The specimen was collected by Sahr Mian, Maarten Christenhusz, Ilia Leitch from the Royal Botanic Gardens Kew. RBG Kew) and Andrew Leitch (from Queen Mary University of London), identified by Maarten Christenhusz and then frozen at –80 °C. The herbarium voucher associated with the sequenced plant is Christenhusz no. 9300 and is deposited in the herbarium of RBG Kew (K) (K001400816).

The genome size was estimated by flow cytometry using the fluorochrome propidium iodide and following the ‘one-step’ method as outlined in Pellicer *et al.* (2021). Specifically for this species, the General Purpose Buffer (GPB) supplemented with 3% PVP and 0.08% (v/v) beta-mercaptoethanol was used for isolation of nuclei ( Loureiro *et al.*, 2007), and the internal calibration standard was *Petroselinum crispum* ‘Champion Moss Curled’ with an assumed 1C-value of 2,200 Mb ( Obermayer *et al.*, 2002).

The workflow for high molecular weight (HMW) DNA extraction at the Wellcome Sanger Institute (WSI) includes a sequence of core procedures: sample preparation; sample homogenisation, DNA extraction, fragmentation, and clean-up. In sample preparation, the dcSalRamo1 sample was weighed and dissected on dry ice ( Jay *et al.*, 2023). For sample homogenisation, leaf tissue was cryogenically disrupted using the Covaris cryoPREP ^®^ Automated Dry Pulverizer ( Narváez-Gómez *et al.*, 2023). HMW DNA was extracted using the Automated Plant MagAttract v2 protocol ( Todorovic *et al.*, 2023a). HMW DNA was sheared into an average fragment size of 12–20 kb in a Megaruptor 3 system with speed setting 30 ( Todorovic *et al.*, 2023b). Sheared DNA was purified by solid-phase reversible immobilisation ( Strickland *et al.*, 2023): in brief, the method employs a 1.8X ratio of AMPure PB beads to sample to eliminate shorter fragments and concentrate the DNA. The concentration of the sheared and purified DNA was assessed using a Nanodrop spectrophotometer and Qubit Fluorometer and Qubit dsDNA High Sensitivity Assay kit. Fragment size distribution was evaluated by running the sample on the FemtoPulse system.

Protocols developed by the WSI Tree of Life core laboratory are publicly available on protocols.io ( Denton *et al.*, 2023).

### Sequencing

Pacific Biosciences HiFi circular consensus DNA sequencing libraries were constructed according to the manufacturers’ instructions. DNA sequencing was performed by the Scientific Operations core at the WSI on a Pacific Biosciences SEQUEL II instrument. Hi-C data were also generated from leaf tissue of dcSalRamo1 using the Arima2 kit and sequenced on the Illumina NovaSeq 6000 instrument.

### Genome assembly, curation and evaluation

Assembly was carried out with Hifiasm ( Cheng *et al.*, 2021) and haplotypic duplication was identified and removed with purge_dups ( Guan *et al.*, 2020). The assembly was then scaffolded with Hi-C data ( Rao *et al.*, 2014) using YaHS ( Zhou *et al.*, 2023). The assembly was checked for contamination and corrected as described previously ( Howe *et al.*, 2021). Manual curation was performed using HiGlass ( Kerpedjiev *et al.*, 2018) and PretextView ( Harry, 2022). The organelle genomes were assembled using OATK ( Zhou, 2023).

A Hi-C map for the final assembly was produced using bwa-mem2 ( Vasimuddin *et al.*, 2019) in the Cooler file format ( Abdennur & Mirny, 2020). To assess the assembly metrics, the *k*-mer completeness and QV consensus quality values were calculated in Merqury ( Rhie *et al.*, 2020). This work was done using Nextflow ( Di Tommaso *et al.*, 2017) DSL2 pipelines “sanger-tol/readmapping” ( Surana *et al.*, 2023a) and “sanger-tol/genomenote” ( Surana *et al.*, 2023b). The genome was analysed within the BlobToolKit environment ( Challis *et al.*, 2020) and BUSCO scores ( Manni *et al.*, 2021; Simão *et al.*, 2015) were calculated.

Table 3 contains a list of relevant software tool versions and sources.

**Table 3.** Software tools: versions and sources.

Software tool	Version	Source
BlobToolKit	4.1.7	https://github.com/blobtoolkit/blobtoolkit
BUSCO	5.3.2	https://gitlab.com/ezlab/busco
Hifiasm	0.16.1-r375	https://github.com/chhylp123/hifiasm
HiGlass	1.11.6	https://github.com/higlass/higlass
Merqury	MerquryFK	https://github.com/thegenemyers/MERQURY.FK
MitoHiFi	2	https://github.com/marcelauliano/MitoHiFi
OATK	0.1	https://github.com/c-zhou/oatk
PretextView	0.2	https://github.com/wtsi-hpag/PretextView
purge_dups	1.2.3	https://github.com/dfguan/purge_dups
sanger-tol/genomenote	v1.0	https://github.com/sanger-tol/genomenote
sanger-tol/readmapping	1.1.0	https://github.com/sanger-tol/readmapping/tree/1.1.0
YaHS	1.2a.2	https://github.com/c-zhou/yahs

### Wellcome Sanger Institute – Legal and Governance

The materials that have contributed to this genome note have been supplied by a Darwin Tree of Life Partner. The submission of materials by a Darwin Tree of Life Partner is subject to the **‘Darwin Tree of Life Project Sampling Code of Practice’**, which can be found in full on the Darwin Tree of Life website here. By agreeing with and signing up to the Sampling Code of Practice, the Darwin Tree of Life Partner agrees they will meet the legal and ethical requirements and standards set out within this document in respect of all samples acquired for, and supplied to, the Darwin Tree of Life Project.

Further, the Wellcome Sanger Institute employs a process whereby due diligence is carried out proportionate to the nature of the materials themselves, and the circumstances under which they have been/are to be collected and provided for use. The purpose of this is to address and mitigate any potential legal and/or ethical implications of receipt and use of the materials as part of the research project, and to ensure that in doing so we align with best practice wherever possible. The overarching areas of consideration are:

•      Ethical review of provenance and sourcing of the material

•      Legality of collection, transfer and use (national and international)

Each transfer of samples is further undertaken according to a Research Collaboration Agreement or Material Transfer Agreement entered into by the Darwin Tree of Life Partner, Genome Research Limited (operating as the Wellcome Sanger Institute), and in some circumstances other Darwin Tree of Life collaborators.

## Data Availability

European Nucleotide Archive: *Salicornia ramosissima*. Accession number PRJEB61611; https://identifiers.org/ena.embl/PRJEB61611 ( Wellcome Sanger Institute, 2023). The genome sequence is released openly for reuse. The *Salicornia ramosissima* genome sequencing initiative is part of the Darwin Tree of Life (DToL) project. All raw sequence data and the assembly have been deposited in INSDC databases. The genome will be annotated using available RNA-Seq data and presented through the Ensembl pipeline at the European Bioinformatics Institute. Raw data and assembly accession identifiers are reported in Table 1.

## Author information

Members of the Royal Botanic Gardens Kew Genome Acquisition Lab are listed here: https://doi.org/10.5281/zenodo.4786680.

Members of the Plant Genome Sizing collective are listed here: https://doi.org/10.5281/zenodo.7994306.

Members of the Darwin Tree of Life Barcoding collective are listed here: https://doi.org/10.5281/zenodo.4893703.

Members of the Wellcome Sanger Institute Tree of Life Management, Samples and Laboratory team are listed here: https://doi.org/10.5281/zenodo.10066175.

Members of Wellcome Sanger Institute Scientific Operations: Sequencing Operations are listed here: https://doi.org/10.5281/zenodo.10043364.

Members of the Wellcome Sanger Institute Tree of Life Core Informatics team are listed here: https://doi.org/10.5281/zenodo.10066637.

Members of the Tree of Life Core Informatics collective are listed here: https://doi.org/10.5281/zenodo.5013541.

Members of the Darwin Tree of Life Consortium are listed here: https://doi.org/10.5281/zenodo.4783558.
