# Supplementary material for: The genome sequence of purple glasswort, Salicornia ramosissima Woods (Amaranthaceae)
Source: Wellcome Open Res. 2024 May 15;9:257. [Version 1] doi: 10.12688/wellcomeopenres.21552.1 (PMC11474152; doi:10.12688/wellcomeopenres.21552.1)
Supplement: Peer review history

# Reviewer report: Reviewer response for version 1

**DOI:** 10.21956/wellcomeopenres.23823.r100757

**Published:** 2024-10-14

**Authors:** Maria Cristina Duarte

**Affiliations:** 1 Universidade de Lisboa, Lisbon, Lisbon, Portugal

This genome note reports the assembly and annotation of the genome of *Salicornia ramosissima *(Amaranthacea), a halophytic species found along the Atlantic and North Sea coasts of Europe.

 The manuscript explains well the motivation behind sequencing the genome of this taxa (for example, its uses as food for humans and animals and its agricultural potential due to its ability to grow in salty habitats, making it a valuable resource for food security), but the need for genomic efforts to unravel the complex taxonomy of the *Salicornia europaea* aggregate could be further emphasised. In fact, *Salicornia ramosissima* belongs to a complex genus and its taxonomic position is not consensual; some authors and reference databases, such as Plants of the World Online (POWO), place it as a synonym of *Salicornia europaea*; moreover, according to IPNI, *Salicornia ramosissima* J.Woods, Bot. Gaz. (London) 3(27): 29 (1851) is an invalid name. On the other hand, World Flora Online (WFO) accepts this taxa. Further taxonomical clarification of the species under study would be much appreciated.

 The protocols used are publicly available and the manuscript describes the materials and methods in detail.

 However, the herbarium voucher - K001400816 (Christenhusz nº 9300) - associated with the sequenced plant and referred to as deposited in the herbarium of RBG Kew (K) is not (yet?) available online in the Kew Data Portal. Moreover, a link to the herbarium specimen will be very useful.

 The data sets are clearly presented and links to interactive tools are also available.

**Some minor comments:**

- Amaranthaceae instead of Chenopodiaceae (in Abstract and in Species taxonomy)
- Italic font is missing in some genus / species names (in Keywords and Species taxonomy)
- According to POWO and WFO *Sarcocornia* is a synonym for *Salicornia* (in the Background).
- *S. ramosissima* and not *S. ramoisissima *(in the Background).

Are sufficient details of methods and materials provided to allow replication by others?

Yes

Is the rationale for creating the dataset(s) clearly described?

Partly

Are the datasets clearly presented in a useable and accessible format?

Yes

Are the protocols appropriate and is the work technically sound?

Yes

Reviewer Expertise:

Plant systematics.

I confirm that I have read this submission and believe that I have an appropriate level of expertise to confirm that it is of an acceptable scientific standard.

# Reviewer report: Reviewer response for version 1

**DOI:** 10.21956/wellcomeopenres.23823.r100759

**Published:** 2024-10-07

**Authors:** Wentao Sheng

**Affiliations:** 1 NanChang Normal University, Nanchang, Jiangxi, China

Dear authors,

 Thank you for submitting this manuscript. The report of the genome from *Salicornia ramosissima *has scientific value. But I have two questions to be resolved:

1. There were only reported genomes, but no further analysis was conducted out.
2. The style is chaotic, and it needs to be scientific standard, then it will be understood easily.

Are sufficient details of methods and materials provided to allow replication by others?

Yes

Is the rationale for creating the dataset(s) clearly described?

Yes

Are the datasets clearly presented in a useable and accessible format?

Yes

Are the protocols appropriate and is the work technically sound?

Yes

Reviewer Expertise:

plant breeding and genome

I confirm that I have read this submission and believe that I have an appropriate level of expertise to confirm that it is of an acceptable scientific standard, however I have significant reservations, as outlined above.

# Reviewer report: Reviewer response for version 1

**DOI:** 10.21956/wellcomeopenres.23823.r97881

**Published:** 2024-09-17

**Authors:** Abhisek Chakraborty

**Affiliations:** 1 Johannes Gutenberg University of Mainz, Mainz, Germany

The authors reported a chromosome-scale genome assembly of *Salicornia ramosissima *in this data note with impressive assembly statistics. The first genome sequence from the *Salicornia *genus will be valuable for the research community.

**Suggestions:**

1. Report the LAI (LTR Assembly Index) score for the genome assembly.
2. Perform the genome annotation and report the statistics, including the BUSCO value for the gene set.

Are sufficient details of methods and materials provided to allow replication by others?

Yes

Is the rationale for creating the dataset(s) clearly described?

Yes

Are the datasets clearly presented in a useable and accessible format?

Yes

Are the protocols appropriate and is the work technically sound?

Yes

Reviewer Expertise:

Genome assembly, Evolutionary Genomics

I confirm that I have read this submission and believe that I have an appropriate level of expertise to confirm that it is of an acceptable scientific standard, however I have significant reservations, as outlined above.
